# Self-medication dilemma in dengue fever

**DOI:** 10.1016/j.puhip.2022.100298

**Published:** 2022-07-11

**Authors:** Muhammad Arslan Khan, Muhammad Junaid Tahir, Muhammad Atif Ameer, Rana Azhar Nawaz, Muhammad Sohaib Asghar, Ali Ahmed

**Affiliations:** a Department of Pharmaceutical Sciences, University of Lahore Teaching Hospital, Lahore, Pakistan; b Lahore General Hospital, Lahore, Pakistan; c University Hospitals Bristol and Weston NHS Foundation Trust, Weston-Super Mare, UK; d Dow University of Health Sciences–Ojha Campus, Karachi, Pakistan; e School of Pharmacy, Monash University, Bandar Sunway, Malaysia

**Keywords:** Dengue, Fever, Self-medication, Surveillance, Control

## Abstract

This paper focuses on the trends of self-medication practices in treating symptoms that may lead to fatal complications in dengue. As dengue is a viral infection with increasing incidence, decision regarding its treatment is mostly affected by public health believes and practices to self-treat the condition by different home remedies, over-the-counter (OTC) drugs or using outdated prescription drugs that proved beneficial in the past experience. Poverty, lack of education, and poor access to health facilities pave the way for making such decisions. Future complications can be averted by raising awareness, counseling the patients and dispensing of pharmaceuticals with strict monitoring.

Dengue is a self-limited, mosquito-borne, systemic, viral disease that spreads by the bite of infected Aedes mosquitos. It is a rapidly emerging pandemic-prone viral disease in many parts globally. In comparison with 50 years ago, the worldwide incidence of dengue has risen 30-fold [1]. The increased incidence of dengue can be associated with uncontrolled urbanization, climate change, humidity, and many other environmental as well as social health systems. The infection is caused by one of the four related dengue virus serotypes (DENV-1, DENV-2, DENV-3, and DENV-4), manifesting symptoms such as fever, nausea, vomiting, rash, frontal headache, myalgia, and arthralgia [2]. The clinical spectrum of the disease ranges from asymptomatic to severe fatal outcomes. At the onset of infection, the symptoms are presumed to be mild, and people typically try to treat them with local herbs or over-the-counter (OTC) drugs [3]. Decisions regarding treatment and seeking health care after the onset of symptoms is affected by a number of factors, including knowledge about dengue, socioeconomic status, lifestyle, and quality of healthcare service [4].

Self-medication is a significantly common practice globally, with prevalence of 32.5–81.5% [5]. It involves the use of drugs to treat symptoms or disorders that are self-recognized, entailing occasional or continued use of drugs prescribed in an old or outdated prescription issued by the physician and irrational use of OTC drugs [6]. Personal health beliefs and practices are among the most important influencing factors involved in treatment decisions [4]. In many cases, people consider seeking care from medical health facilities as a last resort and initially try to treat the symptoms on their own by self-medication, home-remedies, or alternative medicine [3,7]. This trend of self-medication and delay in approaching medical care facilities while fighting dengue can lead to potentially lethal complications. In infectious diseases like dengue, self-medication is a risk factor for fatality. In many studies, mortality due to dengue has been associated with the delay in seeking health care, where appropriate diagnosis and treatment have been given after four or five days of fever in comparison to those who were treated by healthcare professionals in the first three days and recovered [2,8]. Mortality due to dengue in children also depends on the care-seeking behavior of parents. A report was documented on a case of misdiagnosis of dengue symptoms with that of malaria leading to the death of a patient with self-treatment [6].

Considering the barriers promoting the trend of self-medication have highlighted important areas that need focus and improvement. Poor health-seeking behavior can be attributed to illiteracy and poverty in low- and middle-income countries (LMICs) [9]. Inadequate knowledge about the disease leads to the low practice of prevention, in contrast to high education levels that are significantly related to increased practice of prevention from dengue [10]. Low socioeconomic status of patients who are already living a hand-to-mouth life considers treatment from medical care services an additional burden of cost. Individuals living in rural areas find it difficult to access medical facilities as scarce means of transport, poor roads, and traveling costs divert their health care seeking decision towards the use of herbs and self-medication. Lack of patient counseling, lack of access to physicians, and hesitation among patients to consult their condition with physicians can be considered as factors nurturing the trend of self-medication. Refilling of an outdated prescription, poor implementation of the regulation of drug dispensing and advertising, influence from pharmaceutical industries incentives as well as irrational use of OTC drugs are important areas to improve in order to decrease the practice of self-treatment.

Healthcare professionals can play an important role in raising awareness about dengue, disease prevention, and treatment guidelines. Providing patients with the correct information regarding their treatment and counseling them can help prevent misunderstanding about their medications. Strict regulatory control on dispensing of drugs and a trained workforce of pharmacists can help achieve the goal. As social media has become an essential part of survival in today's world, awareness about the risks of self-medication and its consequences in dengue using the internet tool can prove to be a constructive step. A step towards changing the mindset of people preferring self-medication in dengue can help decrease complications.

## Declaration of interests

The authors declare that they have no known competing financial interests or personal relationships that could have appeared to influence the work reported in this paper.

## Financial Support

No financial support was acquired for this article.

## Conflicts of interest

The authors declare no conflict of interest.
